# Supplementary material for: Effect of diabetes on incidence of peritoneal dialysis-associated peritonitis
Source: PLoS One. 2019 Dec 12;14(12):e0225316. doi: 10.1371/journal.pone.0225316 (PMC6907849; doi:10.1371/journal.pone.0225316)
Supplement: Attachment — Submitted filename: Response to Reviewers.docx [file pone.0225316.s001.docx]

**Responses to Comments from the Reviewers**

#1: This article is interesting because it evaluate the effect of diabetes on PD peritonitis over a period of more than 3 decades.

However there are still some problems to accept for publish.

Major

You divided 373 PD patients into 3 groups, according to era. The observation period in 3 groups is different from 7 to 13 years, Era means Period 1 (single bag system), Period 2 (twin bag system), and Period 3 (biocompatible solution).

Is there any article which uses similar era classification? If your original classification, could you mention the history of these development in PD shortly.

**Response:** We have previously reported changes in the clinical features of PD-associated peritonitis using 527 patients who initiated PD at our hospital between January 1980 and December 2012 (16).

As for period classifications, we have revised the sentences in the Discussion as shown below. We divided patients into the three groups according to PD device and solution use. In Japan, the twin-bag system was released from 1992 to 1993 and biocompatible solutions were released from 2000 to 2004.

Our period classification corresponded to the paper by Kitterer et al from German. That paper analyzed 351 adult patients with peritonitis from 1979 to 2014, divided into three time periods (18).

You did not mention about the progress of PD devices such as sterlile system. These devices has been developed mainly to reduce the risk of peritonitis. It is better for you to include the progress of PD devices in era classification.

**Response:** Thank you for these helpful comments. We have added the following sentences In the Subjects and Methods section: “In Japan, sterile systems came into use from 1993 to 1994. This term overlapped with Period 2. However, not every patient used a sterile system, unlike the twin-bag system and biocompatible solution.” As such, we thought did not consider applying use of a sterile system was suitable in era classification.

It seems that we often use device for patients who are empirically risky, for Pariod 3, is there any differences in device usage rate between DM and non DM patients?

**Response:** This study was a post-hoc study of a previously reported cohort study. Therefore, further research was difficult.

Patients tended to start PD without automated disconnecting systems until they developed PD-associated peritonitis, or experienced touch contamination or visual disorders.

Unfortunately, we were not able to research usage rates of connecting systems.

I think it is difficult to explain the result by only biocompatible PD solution.

**Response:** In line with your suggestion, our previous thesis reported that the use of biocompatible solution did not reduce the risk of PD-associated peritonitis.

You mentioned the contribution of icodextrin in discussion, even now some icodextrin are not yet biocompatible neutral solution. If you want to describe the contribution of icodextrin, you should show that you use only biocompatible icodextrin in period 3. Otherwise this phrase should be deleted from the discussion.

**Response:** Thank you for pointing out this issue. As you mentioned, icodextrin and biocompatible solution are not equivalent concepts. We have therefore deleted this sentence from the Discussion section.

The progress of devices seems to have changed the cause of peritonitis from touch contamination to exit and tunnel infection, and intaraperitoneal bacterial translocation, could you show the data on transmission of pathogenic bacteria? If difficult, could you consider only recent period 3 peritonitis whether you can show there was no significant difference between DM and non DM group?

**Response:** Thank you for raising this issue. *Staphylococcus* species predominated in the DM group. However, no statistical differences were evident between DM and NDM groups for the other microorganisms throughout the three periods. We have added mention of this in the revised text.

Minor

1) Could you show the approval number of the ethics committee of Jikei University Hospital.

**Response:** The approval number from the ethics committee at Jikei University Hospital is 30-295(9316). We have provided this information in the revised text.

2) Table 1

What about patients who are observed over both period? Is there a total of 373 patients, or is there no overlap?

**Response:** We have added the following sentence to the Subjects and Methods section ‘When patients used several devices and peritoneal dialysis solutions, we allocated them to the group that they used for more than half of the duration of PD therapy.’

What does PD duration mean in Table 1?

**Response:** ‘PD duration’ was defined as the duration from initiation of PD therapy to the study observation period. We have clarified the definition of PD duration in the revised text.

It is better to show the number of total patient-year in each period.

**Response:** Thank you for pointing this out. We have included total patient-years in Table 1.

Duration of peritonitis is not shown in Table 1. Is there any difference between DM and non DM in each period?

Especially in period 3, the incidence of peritonitis became not significant in both group, what about the duration of peritonitis?

**Response:** No difference in duration of peritonitis was seen between DM and NDM for any periods. We have added the duration of peritonitis in Table 1.

What about the difference in both group about the quality of peritonitis such as simple peritonitis or intractable peritonitis to continue PD (for example MRSA peritonitis, Tuberculosis peritonitis, NTM)?

**Response:** *Staphylococcus* species predominated in DM group. However, regarding *MRSA* and *MRSE*, no significant differences were seen between DM and NDM groups. Only one case of *NTM* was seen in the NDM group for Period 3.

3) Figure 2

How do you define with peritonitis free time? How do you evaluate the second or more peritonitis in same patient? Kaplan Meir Curve seems to deal with only 1st peritonitis in each period considering the number of each period.

**Response:** Peritonitis-free time was defined as the time from start of PD to first occurrence of PD-associated peritonitis. We counted the total number of episodes of PD-associated peritonitis in Table 1.

Regarding Figure 1, we used only first peritonitis, due to the nature of the Kaplan-Meier curve.

Reviewer #2: The authors conducted an analysis based on abundant data unique to institutions that have played a precursor role in Japanese peritoneal dialysis therapy, and in recent years there is no disadvantage in the prevalence of PD-related peritonitis even in diabetic patients.

This study provides extremely useful information for the selection of dialysis methods for diabetic patients. However, some comments should be raised as follows.

1 ISPD guidelines recommend that the parameters monitored should include rates of specific organisms, and the antimicrobial susceptibilities of the infecting organisms.

In diabetic patients, refractory peritonitis or relapsing, recurrent, and repeat peritonitis may affect the results of this study, so the infecting organisms and the frequency of refractory peritonitis, or relapsing, recurrent, and repeat peritonitis should be mentioned.

**Response:** We have mentioned causative organisms of PD-associated peritonitis in the Results and Discussion sections, although we cannot explain antibiotics and refractory, relapsing, recurrent, and repeat peritonitis, as we did not have these data.

We compared peritonitis occurring once or several times between DM and NDM groups for the 3 periods. No significant association was seen between both groups for any period (Period 1 P=0.27, Period 2 p=1.00, Period 3 p=0.51).

2. Exit-site and catheter-tunnel infections are major predis- posing factors to PD-related peritonitis. It should be mentioned whether there were changes in each period of disconnect systems, teaching PD patients and their caregivers, catheter exit site care, and systemic prophylactic antibiotics administration prior to catheter insertion.

**Response:** We used a single-bag system in Period 1, and a twin-bag system after that. Although sterile systems started to be used from Period 2, not every patient used these.

We have revised sentences in the Discussion section as follows. The ISPD recommended teaching PD patients and caregivers about appropriate catheter exit care, and administration of suitable prophylactic antibiotics (22). Although we unfortunately did not have these data, we tried to observe ISPD guidelines. Patients and caregivers were taught techniques for PD when they initiated PD and developed PD-associated peritonitis.

Patients were instructed to disinfect the catheter exit site with povidone iodine. The attending doctor examined the catheter exit site for each patient every month.

Systemic antibiotics were prophylactically administered prior to PD catheter insertion. We used intravenous cefazolin unless clinical contraindications were identified.

3. Since icodextrin is being discussed, changes in the frequency of use of icodextrin should be presented. If information on dialysis fluid is not available even after 2003 in this study, it may be a substitute for discussion based on changes in the frequency of use at other facilities or throughout Japan.

**Response:** Thank you for this helpful comment. We decided to delete the description of icodextrin, as no such mention was included in the thesis.

4. There are no data suggesting improved glycemic control in this study, and the results of this study are not related to discussion.

This is because glycemic control can greatly affect. the results of this study.

In fact, from 1980 to the present, antidiabetic drugs that can be used especially in dialysis patients have been able to add α-glycosidase inhibitors, glinides, DPP-4 inhibitors, and GLP-1 analogs one after another since the days of insulin alone, and insulin itself has changed significantly. These are assumed to have dramatically improved glycemic control.

If data on glycemic control or diabetes treatment for all patients cannot be obtained in this study, the authors should discuss within the available periods, or cite papers on the transition of glycemic control in diabetic patients during PD. At least the transition of treatments for diabetes should be mentioned.

**Response:** We have added the following to the Discussion section “DPP-4 inhibitors and GLP-1 agonists currently play central roles in the treatment of dialysis patients with DM. These drugs were approved between 2009 and 2010 in Japan. Usage of DPP-4 inhibitors was found to significantly improve HbA1c levels and hyperglycemia in patients receiving PD (24,25). Furthermore, GLP-1 agonists significantly decreased average blood glucose levels among diabetic patients undergoing PD (26). Although we did not show data on glycemic control, diabetic patients might have benefitted from these treatments in Period 3.”
